# Biomarkers of optical coherence tomography in evaluating the treatment outcomes of neovascular age-related macular degeneration: a real-world study

**DOI:** 10.1038/s41598-018-36704-6

**Published:** 2019-01-24

**Authors:** Tso-Ting Lai, Yi-Ting Hsieh, Chung-May Yang, Tzyy-Chang Ho, Chang-Hao Yang

**Affiliations:** 1 Department of Ophthalmology, National Taiwan University Hospital, Taipei, Taiwan; 2 Department of Ophthalmology, College of Medicine, National Taiwan University, Taipei, Taiwan; 3 Graduate Institute of Clinical Medicine, College of Medicine, National Taiwan University, Taipei, Taiwan

## Abstract

This study evaluated the characteristic changes in optical coherence tomography (OCT) biomarkers in neovascular age-related macular degeneration (nAMD) treated with anti-vascular endothelial growth factor drugs and their relationship with visual outcomes at 1-year follow-up in a real-world setting. We retrospectively reviewed the medical records of 126 eyes with nAMD treated with either intravitreal ranibizumab or aflibercept, including ophthalmologic examinations and spectral-domain OCT at baseline and months 3, 6, and 12 after first injection. Treatment response of intraretinal cysts (IRCs), subretinal fluid (SRF), and pigment epithelial detachment (PED), and the correlation between best-corrected visual acuity (BCVA) changes and these OCT biomarkers were analyzed. After an average of 5.1 ± 1.5 injections, 33.3% of eyes with PED showed resolution at month 12, a significantly lower proportion than for IRCs (53.8%) or SRF (51.6%). BCVA improvement at 1 year was negatively associated with PED at baseline and with IRCs or PED at month 12. Persistence of IRCs at month 12 was associated with degeneration morphology of IRCs at baseline and non-resolved cysts at month 3 after loading. In conclusions, IRCs and PED are associated with poor visual improvement in nAMD in a real-world setting. Both IRCs and SRF responded better than PED to anti-VEGF therapy.

## Introduction

Age-related macular degeneration (AMD) is one of the most common causes of severe visual loss in middle- and old-aged populations, and often leads to severe impairments in daily life^1,2^. Currently, the first-line treatment for neovascular AMD (nAMD) is intravitreal injection of anti-vascular endothelial growth factor (VEGF) drugs, including bevacizumab, ranibizumab, and aflibercept^3–5^. During both initial evaluation and treatment follow-up for patients with nAMD, optical coherence tomography (OCT) is often used to predict and evaluate the treatment response as well as to guide the treatment^6–12^. Several OCT-based biomarkers, including the central foveal thickness (CFT), the presence of intraretinal cysts (IRCs) or subretinal fluid (SRF), and the presence of pigment epithelial detachment (PED), were found to be associated with initial visual acuity or visual improvements. Schmidt-Erfurth^13^ summarized the usefulness of these OCT-based biomarkers in a recent review, based mainly on the results of post hoc analysis of large randomized control trials (RCTs). However, many of these early studies used time-domain OCT to evaluate the retinal structures and provided less precise evaluation of the retinal details^6,8–10,12^. After spectral-domain OCT (SD-OCT) largely replaced time-domain OCT, more and more studies focused on the effects of specific retinal structural changes on the treatment responses of nAMD patients^11,14–16^. With the use of high resolution images, some OCT findings such as IRCs were further divided into different subtypes, namely degenerative and exudate, which could possibly have different impacts on visual function in nAMD^13,16^. Moreover, a recent study found that there is a considerable difference between RCTs and real-world observational studies regarding the treatment response of nAMD^17^. Despite these observations, there has not been a large study focusing on the usefulness of these high resolution SD-OCT biomarkers in real-world situations, especially of different types of IRCs. In this study, we retrospectively reviewed all nAMD cases treated with either ranibizumab or aflibercept at a tertiary referral center in Taiwan and evaluated the treatment outcomes along with the OCT biomarkers in these patients to further evaluate the predictive value of these OCT findings in a real-world setting.

## Results

**Figure 1:**
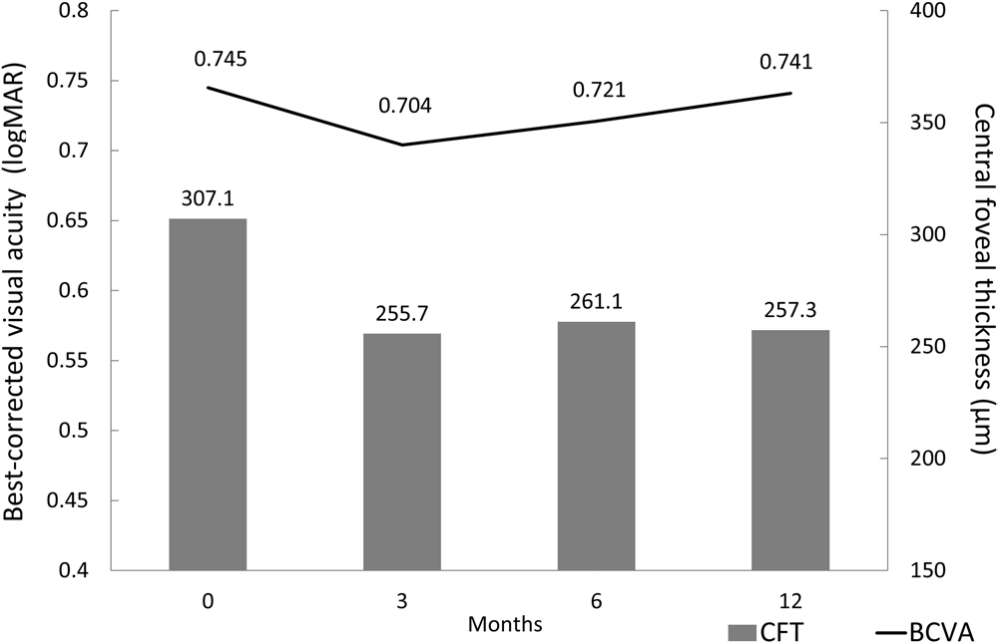
Mean best-corrected visual acuity (BCVA) and central foveal thickness (CFT) at each time point. There were no statistical differences in BCVA between any time points including baseline and months 3, 6, and 12 (*P* > 0.05 for all). The CFT values at months 3, 6, and 12 were significantly less than the baseline value (*P* < 0.001 for all), but the values did not differ between any pair of post-treatment time points (*P* > 0.05 for all).

In total, we included 126 eyes in our study. The subjects were 81 men and 45 women, and the average age was 75.1 ± 9.6 years old. After an average of 5.1 ± 1.5 injections in 1 year, the best-corrected visual acuity (BCVA) remained unchanged, from 0.745 ± 0.325 (Snellen = 20/100 to 20/125) at baseline to 0.741 ± 0.497 (20/100 to 20/125) at month 12 (*P* = 0.947). In contrast, the CFT was reduced significantly, from 307.1 ± 90.8 µm at baseline to 257.3 ± 108.4 µm at month 12 (*P* < 0.001). The mean BCVA and CFT at each time point during the study period are shown in Fig. 1. There were no significant differences in BCVA between baseline and month 3, 6, or 12 (*P* > 0.05 for all). The CFT at month 3, 6, and 12 were all significantly less than at baseline (*P* < 0.001 for all), but did not differ significantly from each other (*P* > 0.05 for all comparisons).

### Factors associated with baseline BCVA

We used linear regression to evaluate possible predicting factors for baseline BCVA and found that the presence of IRC, PED, disrupted external limiting membrane (ELM) and ellipsoid zone (EZ) at initial presentation, as well as a greater CFT at baseline, were associated with poor initial visual acuity (coefficients: 0.170, 0.143, 0.164, 0.335, 0.001; *P* = 0.006, 0.015, 0.004, <0.001, 0.020, respectively). The results are summarized in Table 1. Age, sex, and the presence of epiretinal membrane (ERM) or SRF were not associated with baseline BCVA. After adjusting for age, sex, and baseline CFT in a multiple regression analysis, the presence of PED, disrupted ELM and EZ, and greater baseline CFT were still significantly associated with baseline visual acuity (*P* = 0.022, 0.003, <0.001, 0.024, respectively), while the presence of IRC showed a similar trend but with borderline significance (*P* = 0.053).

**Table 1:** Regression analysis for predicting factors associated with baseline visual acuity in neovascular age-related macular degeneration.

	Coefficient	*P*-value	Adjusted for age, sex, CFT	*P*-value
Age	0.005	0.112	0.004	0.142
Sex (male = 1)	−0.035	0.566	−0.022	0.709
Baseline CFT	0.001	**0.020**	0.001	**0.024**
ERM at baseline	−0.120	0.179	−0.139	0.127
SRF at baseline	−0.014	0.833	−0.015	0.814
IRC at baseline	0.170	**0.006**	0.130	0.053
PED at baseline	0.143	**0.015**	0.133	**0.022**
Disrupted ELM at baseline	0.164	**0.004**	0.171	**0.003**
Disrupted EZ at baseline	0.335	**<0.001**	0.345	**<0.001**

CFT = central foveal thickness; ELM = external limiting membrane; ERM = epiretinal membrane; EZ = ellipsoid zone; IRC = intraretinal cyst; PED = pigment epithelial detachment; SRF = subretinal fluid. Bold text indicates a statistical significance with a p-value less than 0.05.

### Factors associated with BCVA changes at 1 year

In linear regression analyses, we found that the BCVA change at 1 year was significantly associated with the presence of PED or disrupted ELM at baseline (coefficient = 0.167, 0.182; *P* = 0.031, 0.020, respectively) and the presence of IRC or PED at month 12 (coefficients = 0.369, 0.236; *P* < 0.001, *P* = 0.002, respectively). Other factors such as age, sex, the presence of ERM or SRF (at baseline or at month 12), disrupted EZ at baseline, drug type, and total intravitreal injection numbers were not associated with final visual improvement (Table 2). In the multiple regression analysis, poor baseline visual acuity, greater CFT at presentation, presence of PED or disrupted ELM at baseline, and presence of IRC or PED at month 12, were all significantly correlated with less BCVA improvement at month 12, after adjustment for age, sex, and baseline BCVA (coefficients = −0.271, 0.001, 0.205, 0.236, 0.351, 0.241; *P = *0.028, 0.046, 0.009, 0.003, <0.001, 0.001; respectively).

**Table 2:** Regression analysis for predicting factors associated with change of best-corrected visual acuity at 1 year in neovascular age-related macular degeneration treated with ranibizumab or aflibercept.

	Coefficient	*P*-value	Adjusted for age, sex, baseline BCVA	*P*-value
Age	0.002	0.590	0.003	0.458
Sex (male = 1)	−0.017	0.823	−0.019	0.809
Drug type (aflibercept = 1)	−0.024	0.754	−0.018	0.822
Baseline BCVA	−0.202	0.085	−0.271	**0.028**
Baseline CFT	0.001	0.109	0.001	**0.046**
ERM at baseline	0.117	0.317	0.081	0.504
SRF at baseline	−0.070	0.411	−0.085	0.316
IRC at baseline	0.031	0.714	0.004	0.964
PED at baseline	0.167	**0.031**	0.205	**0.009**
Disrupted ELM at baseline	0.182	**0.020**	0.236	**0.003**
Disrupted EZ at baseline	0.018	0.859	0.107	0.325
ERM at month 12	0.094	0.380	0.066	0.562
SRF at month 12	0.150	0.055	0.116	0.159
IRC at month 12	0.369	**<0.001**	0.351	**<0.001**
PED at month 12	0.236	**0.002**	0.241	**0.001**
Total injections	−0.017	0.513	−0.020	0.428

BCVA = best-corrected visual acuity; CFT = central foveal thickness; ELM = external limiting membrane; ERM = epiretinal membrane; EZ = ellipsoid zone; IRC = intraretinal cyst; PED = pigment epithelial detachment; SRF = subretinal fluid. Bold text indicates a statistical significance with a p-value less than 0.05.

### Treatment responses of OCT biomarkers

All 15 eyes presenting with ERM at baseline showed persistence to month 12. Forty-seven (51.6%) of the 91 eyes with SRF at the baseline OCT exam showed resolution at month 12. A similar proportion of patients with IRCs at baseline showed resolution at month 12 (21 of 39; 53.8%, *P* = 0.818). In contrast, only 25 (33.3%) of the 75 eyes with PED at baseline showed no PED on the final OCT exam. This proportion was significantly lower than the levels of resolution for SRF or IRCs (*P* = 0.018 and *P* = 0.034, respectively). There were no significant differences between ranibizumab and aflibercept regarding the treatment responses of IRCs, SRF, or PED. Twenty-six eyes (51.0%) in the ranibizumab group and 21 eyes (52.5%) in the aflibercept group showed resolution of SRF at month 12 (*P* = 0.886). Intraretinal cysts resolved in 14 (50.0%) eyes in the ranibizumab group and 7 eyes (63.6%) in the aflibercept group (*P* = 0.442). Of the eyes with PED on the baseline OCT exam, 12 (26.6%) of the ranibizumab-treated eyes and 13 (43.3%) of the aflibercept-treated eyes showed resolution of PED at month 12 (*P* = 0.134).

### Association of OCT biomarkers and BCVA response at different time points

**Figure 2:**
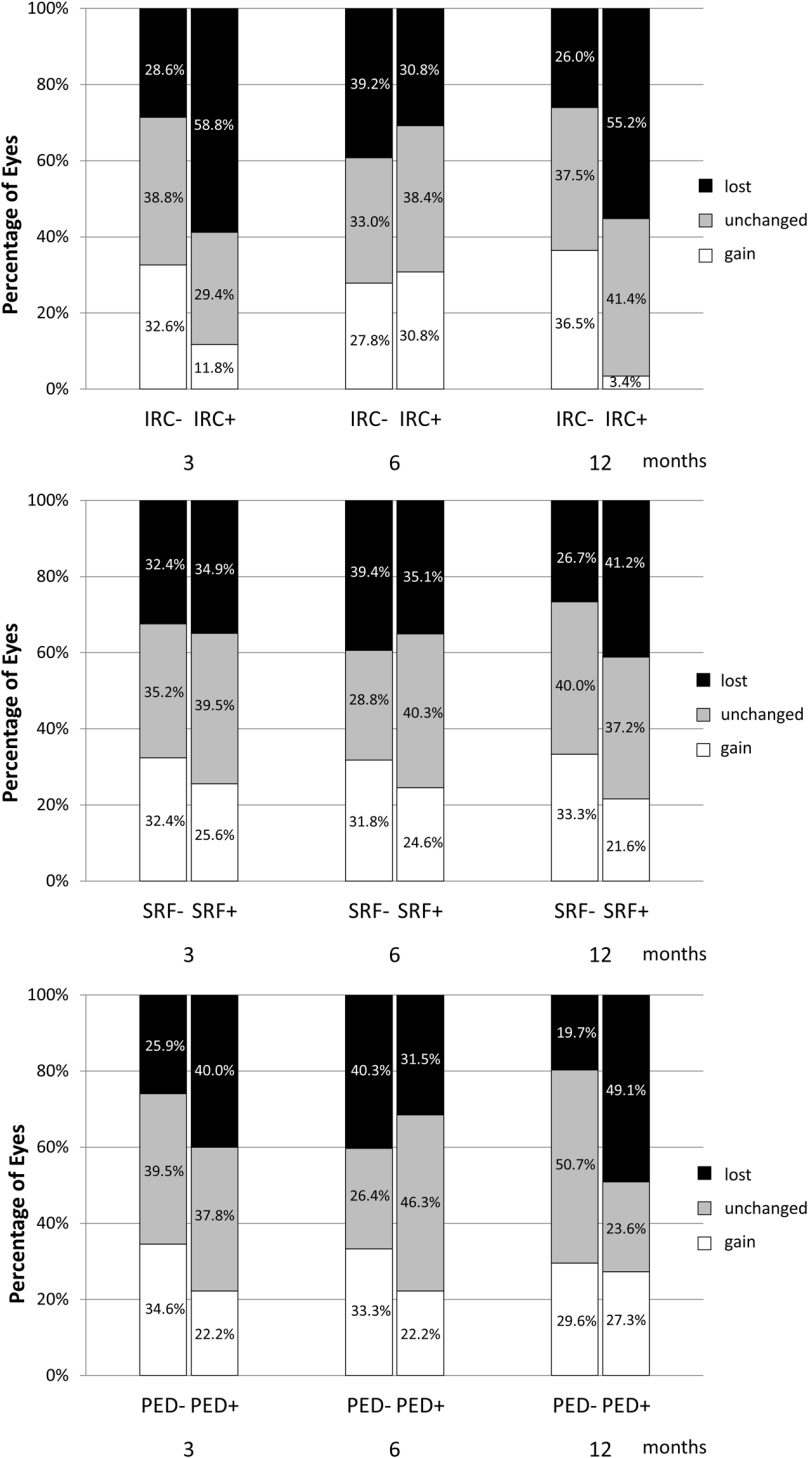
Relationship between optical coherence tomographic (OCT) biomarkers and visual acuity response at each time point. Each OCT biomarker was classified as present or absent at each time point. The treatment response was classified into 3 groups according to the change in logMAR visual acuity: gain (≤−0.2), unchanged (≤0 to >−0.2), and lost (no improvement, >0). (Top) A significant difference in treatment response was found between patients with and without intraretinal cysts (IRCs) at months 3 and 12, but not at month 6. (Middle) No significant differences was found in treatment response at any time point corresponding to presence or absence of subretinal fluid (SRF). (Bottom) The group of patients with pigment epithelial detachment (PED) at month 12 included significantly more cases with lost vision and fewer with unchanged compared with the group without PED, but no differences were found at months 3 or 6.

The associations of OCT biomarkers and BCVA changes at months 3, 6, and 12 are shown in Fig. 2. The BCVA response (classified as gain, unchanged, or lost; ≥10 ETDRS letters gained, >10 but ≥0 letters change, or loss ETDRS letters) was significantly associated with the presence of IRC at month 3, and both IRC and PED at month 12 (chi-square test, *P* = 0.039, *P* < 0.001, and *P* < 0.001, respectively). For patients with IRC at baseline, the BCVA improvement was less in those with persisted IRC at month 12 (0.160 ± 0.379) compared to those with resolved cyst (−0.078 ± 0.329, *P* = 0.048).

### Prediction of persistent IRC from baseline morphology and treatment response at month 3

**Figure 3:**
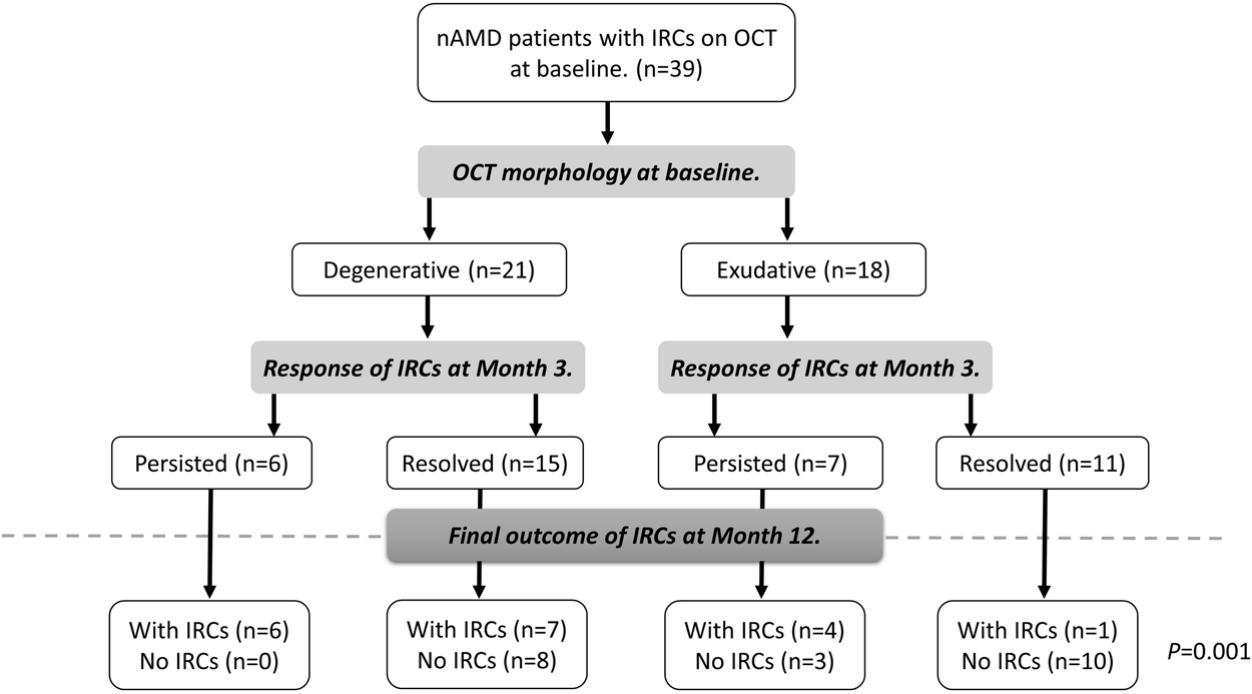
Flowchart illustrating the changes in presence of intraretinal cysts (IRCs) in neovascular age-related macular degeneration patients presenting with IRCs at baseline using 2 checkpoints: the morphology of the cyst at baseline and the response of the IRC at month 3. OCT: optical coherence tomography.

According to our OCT morphological criteria (as described in the methods section), 21 eyes were classified as having cysts with degenerative morphology and the other 18 eyes as having IRC with exudative morphology. We found a significant correlation between the baseline OCT morphology of IRC and the persistence of the cysts at month 12 (*P* = 0.033). Thirteen (61.9%) of the 21 eyes with degenerative morphology showed persistent cysts at month 12, while only 5 (27.8%) of the 18 eyes classified with exudative morphology showed persistent cysts at month 12. In addition to the OCT morphology at baseline, the treatment response of IRCs at month 3 was also strongly associated with the persistence of IRCs at month 12. Ten (76.9%) of the 13 eyes showing a degenerative response (persistent IRCs at month 3) had persistent cysts at month 12, whereas 18 (69.2%) of the 26 eyes with an exudative response (resolved cysts) at month 3 continued to showed no IRC on OCT at month 12 (*P* = 0.006). Taking both OCT morphology and treatment response into consideration gave the highest predictive power for persistence of IRCs at month 12 (*P* = 0.001). All 6 eyes with both degenerative morphology and degenerative treatment response had persistent IRC at month 12, whereas only one of the 11 eyes with both exudative morphology and exudative treatment response had persistent IRC at month 12 (Fig. 3).

### Treatment response at month 3 predicted the final visual improvement

**Figure 4:**
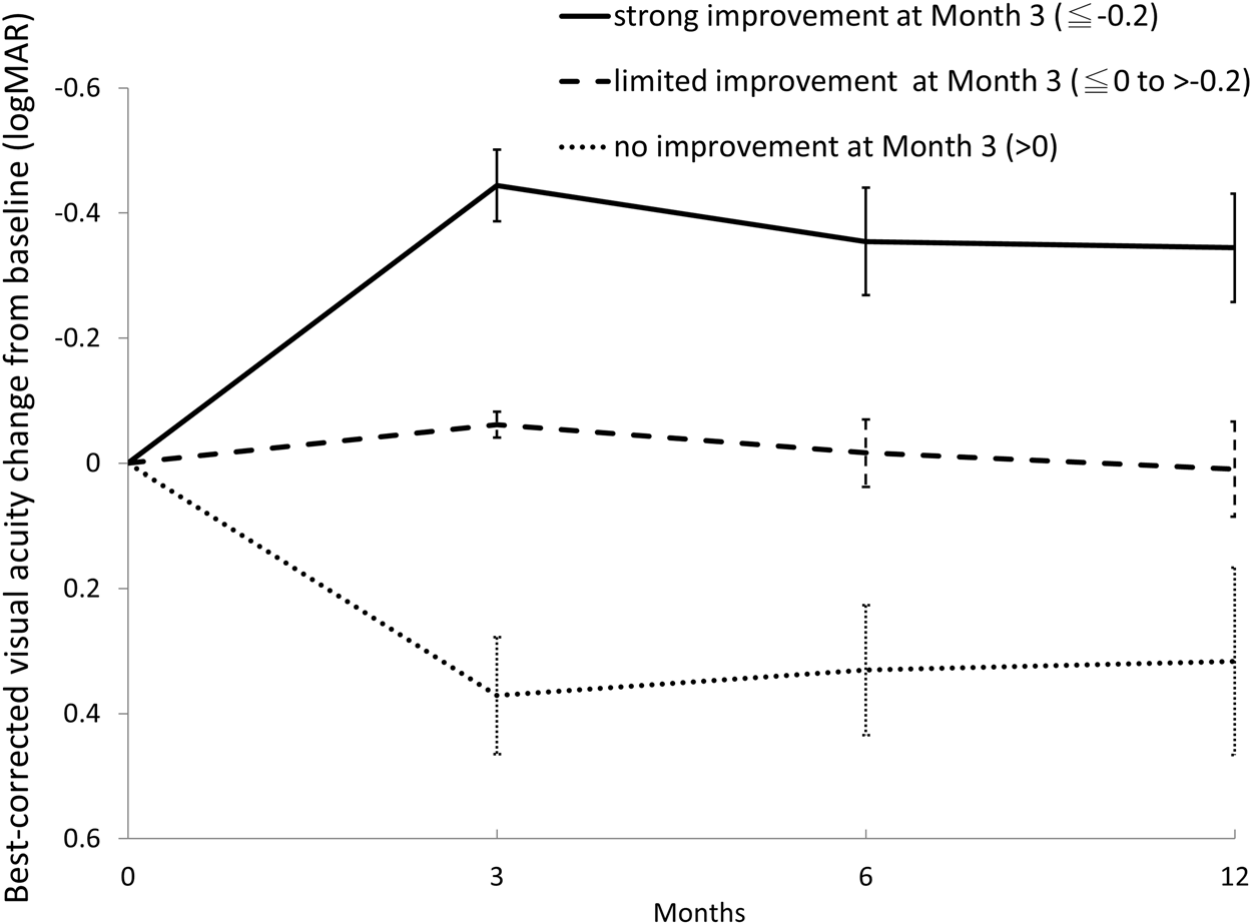
Comparison of best-corrected visual acuity (BCVA) changes from baseline at different time points, split according to treatment response at month 3. The patients were divided into 3 groups according to the BCVA change at Month 3: strong improvement (change in logMAR BCVA ≤−0.2), limited improvement (change in logMAR BCVA ≤0 to >−0.2), and no improvement (change in logMAR BCVA >0). At months 3, 6, and 12 the BCVA improvement was significantly better in the strong improvement group than in the limited improvement group, and both were significantly better than in the no-improvement group (*P* < 0.001 for all comparisons).

At month 3, 37 eyes (29.4%) showed strong improvement in BCVA, 49 eyes (38.9%) showed limited improvement, and 40 eyes (31.7%) showed no improvement. The patients with strong improvement started with worse baseline visual acuity (0.801 ± 0.328; Snellen = 20/125 to 20/160) than the patients in the limited improvement (0.662 ± 0.283, *P* = 0.003; 20/80 to 20/100) and no improvement groups (0.726 ± 0.339, *P* = 0.047; 20/100 to 20/125), but ended up with best BCVA (0.502 ± 0.351; Snellen = 20/63) at month 12 (0.672 ± 0.388 for the limited improved group, 20/80 to 20/100, *P* = 0.083; 1.042 ± 0.567 for the no improvement group, 20/200 to 20/400, *P* < 0.001) groups. The visual acuity gain was greater for the strong improvement group than for the limited improvement group at every time point, and were both better than the no improvement group (Fig. 4, *P* < 0.001 for all).

## Discussion

The real-world results in our study demonstrated a strong correlation between baseline OCT biomarkers and visual function at both baseline and 1 year in patients with nAMD treated with either ranibizumab or aflibercept. A post hoc analysis of the VIEW studies^12^ also found that morphologic features detectable on OCT, such as IRC, SRF, and PED, were significantly associated with baseline visual acuity and BCVA changes at week 52. In their study, they found that IRC at baseline were the most important features associated with both poor baseline visual acuity and lower visual improvement, while SRF at baseline was associated with better BCVA gain, and PED at baseline was associated with reduced BCVA gain. In our study, we also found that baseline PED was an important predictor of BCVA improvement at month 12. Patients showed worse baseline visual acuity and less visual improvement if PED was present before treatment. Simader *et al*. reported on a subanalysis of the EXCITE study^8^ in which they found that PED predicted a poor visual outcome only if combined with IRC or SRF. However, we found a negative effect on visual improvement in patients with PED regardless of these other factors. The presence of PED, especially fibrovascular PED, indicated growth of neovascularization with leakage of fluid, which leads to poor visual function^14,18^. PEDs are also less responsive to anti-VEGF treatment than SRF or IRC in nAMD^9,19^. Fibrovascular PED accounted for 60% of our PED patients, and two-thirds of our PED patients had persistent PED after 12 months. The combination of unfavorable PED subtype and large proportion of persistent PED was responsible for the poor visual improvement related to PED.

The presence of IRC was found to associate with poor baseline visual acuity and reduced visual improvement in other post hoc analyses of data from large RCTs^8,12^. Our real-world study, however, found that although the presence of IRCs at baseline indicated lower baseline BCVA, it was not associated with BCVA improvement at month 12. On the other hand, the presence of IRC at month 12 was a strong indicator for poor visual improvement in our study. Schmidt-Erfurth and Waldstein^13^ classified IRC into two different types, exudative and degenerative, based on differences in morphology and different treatment responses. The degenerative cysts are often small, with underlying RPE atrophy or scarring, whereas the exudative cysts are larger ovoid cysts that responded relative quickly to anti-VEGF treatments^10,13,15,16^. It is possible that both exudative and degenerative IRC were present at baseline in our study. While both types of cysts caused impaired visual acuity at baseline, the exudative cysts responded well to anti-VEGF treatment and resolved during the study period with improved BCVA; in contrast, the degenerative cysts persisted during treatment and resulted in poor visual improvement at month 12. Thus, BCVA improvement at month 12 was only correlated with the presence of IRC at month 12, not with IRC at baseline.

Although the differences between exudative and degenerative IRCs have been discussed in several studies^9,10,16^, most of them were only descriptive, and there are no well-established criteria to differentiate one type from the other in routine clinical settings. In our study, we used the shape and size of cysts, the changes to adjacent structures, and retinal pigment epithelium (RPE) changes, to formulate a more objective criterion for classifying the morphology of IRCs. We also combined both OCT morphology and treatment response to classify these two types of IRCs and found a strong correlation with the final status of the IRCs at month 12, which was significantly associated with the final BCVA improvement. The characteristic OCT morphological features of degenerative IRCs, such as smaller size and square shape, could give us early clues to predict a possible poor response before anti-VEGF treatment begins. However, despite the fact that a significant correlation between baseline IRC morphology and the final appearance of IRCs was found in our study, the morphological criteria are still considered somewhat subjective, and 38% of the cysts with degenerative morphology had resolved at month 12. It was possible that some cysts, although showing “degenerative” morphology on OCT at baseline, were actually “exudative” in nature thus these cysts resolved after treatment. Adding the treatment response after loading injections allowed better predictions of IRC persistence at Month 12. All the eyes with both degenerative OCT morphology and degenerative treatment response showed persistent IRCs at month 12 despite treatment, indicating a chronic course or a refractory disease. These patient may need either more aggressive therapy and modification of treatment modalities if there were other signs of disease activity on OCT or fluorescein angiography, or consider hold treatment if no other signs besides these degenerative cysts were present, since degenerative cysts along was reported not as a sign of active AMD in previous studies^13,16^. When both OCT morphology and the treatment response at month 3 were exudative, only one out of eleven eyes had IRCs at month 12. This could predict a better treatment outcome, meaning that the patient might be a candidate for less frequent follow-up or for a different treatment regimen such as treat-and-extend, although further studies are needed to confirm this inference.

We found that the treatment response after 3 loading injections—not only the changes in IRCs but also the changes in BCVA—could be helpful in predicting the long-term response. Bloch *et al*.^11^ found that the visual acuity at month 3 was the strongest predictor of final BCVA at month 12. In our study, we stratified our patients according to the visual improvement at month 3 and found that differences in treatment responses generally persisted to the final visit. Patients with strong improvement at month 3 ended up with an average improvement of nearly 20 ETDRS letters. On the other hand, those with no improvement at month 3 lost an average of more than 15 letters by the end of our study. The deterioration of visual acuity in the latter group was often secondary to the development of subretinal scars or RPE atrophy during treatment. Based on the result of our study, it would be reasonable to adjust the treatment strategy after 3 loading injections according to the month 3 response, especially for patients with suboptimal improvements, since the same response is likely to persist to month 12 if no changes are made.

The integrity of retinal outer layers, the ELM and EZ, were found to be associated with visual acuities in patients with nAMD^20,21^. Worse visual acuities were found at baseline and after treatment when ELM or EZ were disrupted. However, further studies also found the integrity of EZ could restore after anti-VEGF treatments, and the visual improvement was not associated with disrupted EZ at baseline^22,23^. On the other hand, the ELM had less plasticity and the recovery of EZ occurred when ELM was intact^22^. A recent study also reported a higher incidence of visual loss in patients with disrupted ELM at baseline after aflibercept treatment^24^. In our study, we found only disrupted ELM was associated with less BCVA improvement after treatment, although both ELM and EZ integrity were associated with baseline BCVA. These findings are in accordance with previous reports, and supports the idea that intact ELM is important for EZ restoration and will affect visual improvement.

The limitations of our study include its retrospective nature and possible selection bias in the use of different anti-VEGF agents. Difference in the OCT machines used in our study is also a possible bias, since 6.3% of patient received OCT exam with 2 different machines. Another limitation is the relative small number of cases, especially in subgroup analysis, therefore the results should be interpreted with caution. However, our study reflects real-life conditions in daily practice along with detailed OCT findings obtained from high resolution OCT scans. The average injection numbers in our study, although lower than those in previous clinical trials^4,8,12^, were similar to those of previously reported observational studies^17,25,26^. The differences between the real-world results and RCTs in both treatment frequency and treatment outcomes are important information for physicians in managing their patients in daily practice. Moreover, most observational studies using registration data did not have access to detailed OCT image evaluations and could not analyze the relationship between OCT biomarkers and treatment outcomes in a real-world setting^17,25,26^. Our study, despite a smaller number of cases compared to studies using registration data, provided a detailed analysis using sequential OCT finding, and demonstrated the relationship between OCT biomarkers and treatment outcomes.

In conclusion, our study found similar visual and anatomical outcomes for nAMD patients treated with ranibizumab or aflibercept after similar numbers of injections in a real-world setting. Among the OCT biomarkers that were examined, PED and persistent IRCs were important predictors of poor visual improvement, and we could use baseline OCT morphology and the treatment response after loading injections to differentiate exudative from degenerative IRCs and thereby better predict the persistence of cysts at month 12. These findings could further help predict treatment responses as well guide physicians in adjusting their treatments.

## Methods

### Study Population and Setting

We retrospectively reviewed the clinical charts of all patients who started intravitreal ranibizumab or aflibercept injection for nAMD under the reimbursement of the National Health Insurance (NHI) at National Taiwan University Hospital from January 2014 to February 2016. The criteria for prescribing anti-VEGF drugs for nAMD set by the NHI include: (1) BCVA between 0.05 to 0.5 (decimal, equal to 20/400 to 20/40), (2) fluorescein angiography and OCT showing findings of nAMD without evidence of other causes of neovascularization such as uveitis, myopic choroidal neovascularization, or other secondary choroidal neovascularization, (3) no visible polypoidal lesion on Indocyanine Green angiography (to exclude polypoidal choroidal vasculopathy), (4) no evidence of geographic atrophy or macular scar on either OCT or fluorescence angiography. In addition to the NHI criteria, a minimum follow-up time of 1 year was required for enrollment in our study. Patients were excluded from our study if they had received other treatments for nAMD (including other anti-VEGF agents, steroids, or photodynamic therapy) within 6 months prior to the first injection reimbursed by NHI. Patients who had switched from ranibizumab to aflibercept or vice versa were also excluded. The study adhered to the tenets of the Declaration of Helsinki and was approved by the National Taiwan University Hospital Research Ethics Committee, and adequate informed consent was obtained from all patients.

### Clinical Data Collection

Patient demographic data including age, sex, and eye or eyes involved at baseline were recorded. The BCVA measurements were recorded at baseline as well as at each follow-up visit, including 3, 6, and 12 months after the first injection, and were converted to logMAR scores for calculations. OCT examinations were performed at baseline and at each follow-up visit. Central foveal thickness was obtained using the built-in macular thickness map program with the SD-OCT system (Cirrus^TM^ HD-OCT, Carl Zeiss Meditec, Inc., Dublin, CA, USA; or RTVue^®^ Model-RT100 version 3.5, Optovue, Inc., Fremont, CA, USA).

### Classification of OCT findings

**Figure 5:**
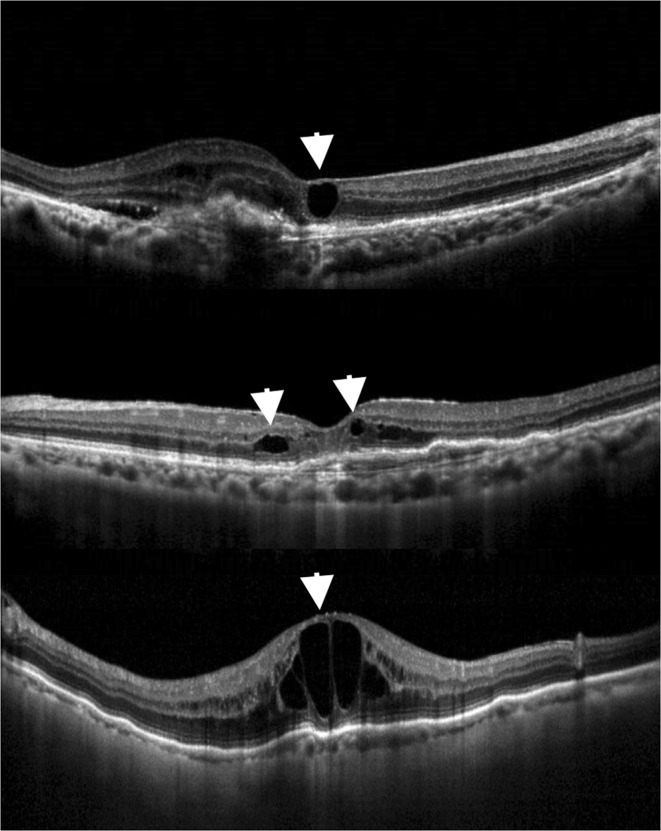
Optical coherence tomography (OCT) images of different types of morphology of intraretinal cysts (IRCs) in age-related macular degeneration. (Top) OCT image showing degenerative morphology of an IRC (arrowhead) with square-shaped contour and alteration of underlying retinal pigment epithelium. (Middle) Another example of degenerative morphology of IRCs (arrowheads) on OCT, demonstrating a small cyst without obvious expansion of the adjacent layered retinal structure (arrowhead on the right) and an IRC with square-shaped contour (arrowhead on the left). (Bottom) Example of exudative IRCs on OCT, showing large cysts along with stretched adjacent retinal tissue and normal retinal pigment epithelium.

All OCT images were reviewed independently by two investigators (C-HY and T-TL) for the presence of ERM, IRC, SRF, and PED. The baseline integrity of ELM and EZ were also evaluated. These findings were defined as present only when the central 3 mm of macula was involved. If an IRC was noted on the baseline OCT exam, it was further classified as exudative or degenerative using criteria derived from previous studies^13,16^. Since there are no well-established criteria for differentiating exudative from degenerative cysts, we looked for the three following findings: (1) alteration of the RPE layer underlying the cyst; (2) a squared-shaped lesion with at least one concave or straight border; (3) small size of the IRC (greatest dimension <125 µm) without obvious expansion of adjacent layered retinal structure. Cysts having as least 2 of these morphological features were classified as having “degenerative morphology”; the rest were classified as having “exudative morphology” (Fig. 5). The PED height was manually measured using a built-in caliper tool, from the most elevated RPE to an ideal RPE line, and PED was defined to be present by the criterion PED height >150 µm. PED lesions were further classified as serous PED if the OCT images showed a homogeneously hyporeflective sub-RPE space, and were otherwise classified as fibrovascular PED.

### Treatment Protocol

The patients received either 0.5 mg of ranibizumab or 2 mg aflibercept for each involved eye. The choice between ranibizumab and aflibercept was based on the physician’s preference. During the first 3 months (the initial loading phase), three consecutive monthly injections were given. Injections given beyond month 3 were considered as additional injections. Additional injections were given based on the clinical findings during each follow-up visit, mainly with an as-needed, *pro re nata* (PRN) approach, at the doctor’s discretion. The type of drug used and the total number of anti-VEGF injections within 1 year were recorded.

### Treatment Response and Grouping

The BCVA and CFT at the 3, 6, and 12-month follow-up visits were compared with the baseline data. The patients were then categorized into 3 groups (strong, limited, and no improvement) based on the visual acuity gain at month 3, as measured in logMAR units. The criteria were:

- Strong improvement: ≤−0.2 logMAR change; equivalent to ≥10 Early Treatment Diabetic Retinopathy Study (ETDRS) letters gained.
- Limited improvement: logMAR change between 0 and −0.2; equivalent to 0 to 10 letters gained.
- No improvement: logMAR change >0; equivalent to <0 letters gained.

### Differentiation of Degenerative Cysts from Exudative Cysts

We used both baseline OCT morphology and the treatment response at month 3 to differentiate degenerative cysts from exudative cysts. The OCT criteria for degenerative morphology was described in previous sections. Those cysts meeting 2 of the 3 criteria were defined as having “degenerative morphology”. For patients presenting with IRCs at baseline, we classified those with resolved cysts at month 3 as showing “exudative responses” and those with persistent cysts at month 3 as showing “degenerative responses”.

### Statistical analysis

Comparisons of BCVA and CFT measurements between baseline and follow-up visits were performed with paired Student’s t-tests. Intergroup comparisons for the subgroup analysis categorized by OCT biomarkers were performed using Student’s t-tests for continuous variables and Chi-square tests for categorical variables. Multiple regression analyses were performed to evaluate the possible predictive factors for baseline visual acuity and final visual improvements at 12 months. For comparisons among groups with different treatment response at Month 3, ANOVAs and post hoc analysis with Bonferroni corrections were used. For baseline visual acuity, the candidate predictive factors including age, sex, baseline CFT, and baseline OCT biomarkers including ERM, SRF, IRC, PED, disrupted ELM and EZ; age, sex and baseline CFT were adjusted in all models. For final visual acuity improvement, the candidate predictive factors including age, sex, baseline visual acuity, baseline CFT, OCT biomarkers at baseline, OCT biomarkers at Month 12, drug type and total number of injections; age, sex and baseline visual acuity were adjusted in all models. The data were analyzed using SPSS software (SPSS 22.0; SPSS Inc., Chicago, IL, USA). A *P* value of less than 0.05 was considered statistically significant.

## References

[1] Yonekawa Y, Miller JW, Kim IK (2015). Age-Related Macular Degeneration: Advances in Management and Diagnosis. Journal of clinical medicine.

[2] Shaikh AH (2015). Cost comparison of intravitreal aflibercept with bevacizumab and ranibizumab for the treatment of wet age-related macular degeneration. Ophthalmic surgery, lasers & imaging retina.

[3] Sarwar S (2016). Aflibercept for neovascular age-related macular degeneration. The Cochrane database of systematic reviews.

[4] Rosenfeld PJ (2006). Ranibizumab for neovascular age-related macular degeneration. The New England journal of medicine.

[5] Heier JS (2012). Intravitreal aflibercept (VEGF trap-eye) in wet age-related macular degeneration. Ophthalmology.

[6] Fung AE (2007). An optical coherence tomography-guided, variable dosing regimen with intravitreal ranibizumab (Lucentis) for neovascular age-related macular degeneration. American journal of ophthalmology.

[7] Holz FG (2011). Safety and efficacy of a flexible dosing regimen of ranibizumab in neovascular age-related macular degeneration: the SUSTAIN study. Ophthalmology.

[8] Simader C (2014). Morphologic parameters relevant for visual outcome during anti-angiogenic therapy of neovascular age-related macular degeneration. Ophthalmology.

[9] Schmidt-Erfurth U, Waldstein SM, Deak GG, Kundi M, Simader C (2015). Pigment epithelial detachment followed by retinal cystoid degeneration leads to vision loss in treatment of neovascular age-related macular degeneration. Ophthalmology.

[10] Bolz M (2010). Morphological and functional analysis of the loading regimen with intravitreal ranibizumab in neovascular age-related macular degeneration. The British journal of ophthalmology.

[11] Bloch SB (2013). Predictors of 1-year visual outcome in neovascular age-related macular degeneration following intravitreal ranibizumab treatment. Acta ophthalmologica.

[12] Waldstein SM (2016). Morphology and Visual Acuity in Aflibercept and Ranibizumab Therapy for Neovascular Age-Related Macular Degeneration in the VIEW Trials. Ophthalmology.

[13] Schmidt-Erfurth U, Waldstein SM (2016). A paradigm shift in imaging biomarkers in neovascular age-related macular degeneration. Progress in retinal and eye research.

[14] Clemens CR (2012). High-resolution optical coherence tomography of subpigment epithelial structures in patients with pigment epithelium detachment secondary to age-related macular degeneration. The British journal of ophthalmology.

[15] Waldstein SM (2016). Correlation of 3-Dimensionally Quantified Intraretinal and Subretinal Fluid With Visual Acuity in Neovascular Age-Related Macular Degeneration. JAMA ophthalmology.

[16] Querques G (2011). Cystoid macular degeneration in exudative age-related macular degeneration. American journal of ophthalmology.

[17] Chong V (2016). Ranibizumab for the treatment of wet AMD: a summary of real-world studies. Eye.

[18] Pauleikhoff D (2002). Pigment epithelial detachment in the elderly. Clinical differentiation, natural course and pathogenetic implications. Graefe’s archive for clinical and experimental ophthalmology = Albrecht von Graefes Archiv fur klinische und experimentelle Ophthalmologie.

[19] Cho HJ (2016). Response of Pigment Epithelial Detachment to Anti-Vascular Endothelial Growth Factor Treatment in Age-Related Macular Degeneration. American journal of ophthalmology.

[20] Oishi A (2010). The significance of external limiting membrane status for visual acuity in age-related macular degeneration. American journal of ophthalmology.

[21] Mathew R, Richardson M, Sivaprasad S (2013). Predictive value of spectral-domain optical coherence tomography features in assessment of visual prognosis in eyes with neovascular age-related macular degeneration treated with ranibizumab. American journal of ophthalmology.

[22] Oishi A (2013). Recovery of photoreceptor outer segments after anti-VEGF therapy for age-related macular degeneration. Graefe’s archive for clinical and experimental ophthalmology.

[23] Kim YM, Kim JH, Koh HJ (2012). Improvement of photoreceptor integrity and associated visual outcome in neovascular age-related macular degeneration. American journal of ophthalmology.

[24] Hata M (2017). Incidence and causes of vision loss during aflibercept treatment for neovascular age-related macular degeneration: One-Year Follow-up. Retina.

[25] Ferreira A (2015). Treatment Frequency and Dosing Interval of Ranibizumab and Aflibercept for Neovascular Age-Related Macular Degeneration in Routine Clinical Practice in the USA. PloS one.

[26] Lotery A, Griner R, Ferreira A, Milnes F, Dugel P (2017). Real-world visual acuity outcomes between ranibizumab and aflibercept in treatment of neovascular AMD in a large US data set. Eye (Lond).

